# A Proposal of a Troposphere Model in a GNSS Simulator for VANET Applications

**DOI:** 10.3390/s21072491

**Published:** 2021-04-03

**Authors:** Mauro Tropea, Angelo Arieta, Floriano De Rango, Francesco Pupo

**Affiliations:** 1 Department of Informatics, Modeling, Electronics and System Engineering (DIMES), University of Calabria, Via P.Bucci 39/c, 87036 Rende, Italy; m.tropea@dimes.unical.it (M.T.); pupo@dimes.unical.it (F.P.); 2 NTT Data, c.da Cutura, Via Spagna 50, 87036 Rende, Italy; angelo.arieta@nttdata.com

**Keywords:** positioning system, VANET, troposphere model, GNSS simulator, GPS receiver

## Abstract

Vehicle positioning is becoming an important issue related to Intelligent Transportation Systems (ITSs). Novel vehicles and autonomous vehicles need to be localized under different weather conditions and it is important to have a reliable positioning system to track vehicles. Satellite navigation systems can be a key technology in providing global coverage and providing localization services through many satellite constellations such as GPS, GLONASS, Galileo and so forth. However, the modeling of positioning and localization systems under different weather conditions is not a trivial objective especially considering different factors such as receiver sensitivity, dynamic weather conditions, propagation delay and so forth. This paper focuses on the use of simulators for performing different kinds of tests on Global Navigation Satellite System (GNSS) systems in order to reduce the cost of the positioning testing under different techniques or models. Simulation driven approach, combined with some specific hardware equipment such as receivers and transmitters can characterize a more realistic scenario and the simulation can consider other aspects that could be complex to really test. In this work, the main contribution is the introduction of the Troposphere Collins model in a GNSS simulator for VANET applications, the GPS-SDR-SIM software. The use of the Collins model in the simulator allows to improve the accuracy of the simulation experiments throughout the reduction of the receiver errors.

## 1. Introduction

The Global Navigation Satellite System (GNSS) refers to a satellite navigation system for providing global coverage thanks to a satellites constellation providing signals from space. It is a geo-radiolocation for terrestrial, marine or air navigation systems [1]. The satellites transmit positioning and timing data to GNSS receivers that use this data to determine their position. Thanks to this global coverage, the receivers that are located anywhere on the Earth’s surface or on the atmosphere, can determine their geographic coordinates by processing the Radio Frequency (RF) signals transmitted by satellites. They use the trilateration operation, in order to obtain its position, as each satellite continuously sends information regarding the ephemeris [2]. Ephemeris are information concerning the position, the clock (timing) and health of the satellites [3] sent through the navigation message that is transmitted by satellites [4]. GNSS performance is evaluated by four principles—Accuracy: evaluating differences between a receiver’s measured and real position, speed or time; Integrity—evaluating a system’s capacity to provide a threshold of confidence and, in the event of an anomaly in the positioning data, an alarm; Continuity: evaluating a system’s ability to function without interruption; Availability, evaluating the percentage of time a signal fulfils the above accuracy, integrity and continuity principles. This performance can be improved by regional satellite-based augmentation systems (SBAS), such as the European Geostationary Navigation Overlay Service (EGNOS), enhancing the GPS accuracy and reliability correcting measurement errors and providing information integrity on signals.

**Figure 1:**
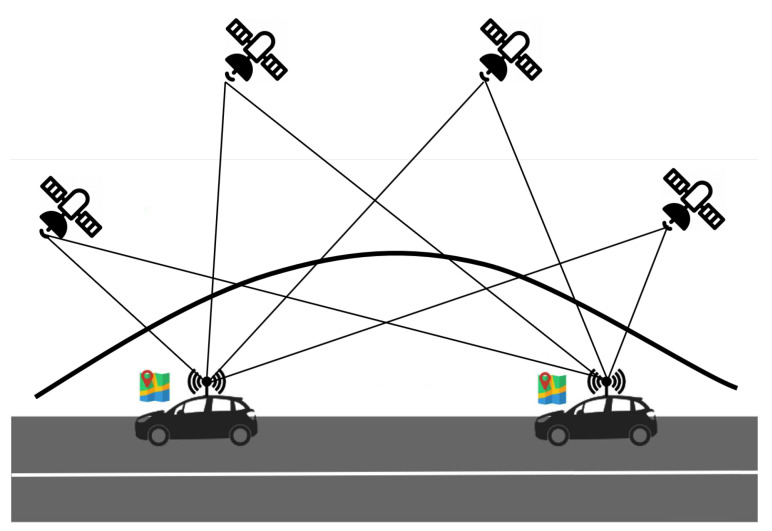
Global Navigation Satellite System (GNSS) in a Vehicular Ad-Hoc Network (VANET) scenario.

Today the navigation systems are considered an integrated part of the Intelligent Transportation System (ITS) and Vehicular Ad-Hoc Network (VANET) system [5]. The vehicles are equipped with GPS receivers and, in the next future, all vehicles will have a GNSS receiver on board [6,7]. In Figure 1 a typical scenario of global position system in a VANET environment is shown.

The vehicles network aims to improve road safety, increase traffic efficiency and save energy giving also attention to the emission issues [8,9], and also different works focus their attention on mechanisms to warn dangerous or emergency situations by exploiting on-board sensors [10], for routing purposes [11] and also using predictive mechanism as in [12,13]. Incoming automatic driving applications are going to require even tighter level of precision and security in order to guarantee the high required standard. The first sector using detailed GNSS integrity solutions is that of aviation.

In this paper, taking as reference scenario the field of vehicles’ applications, we propose the integration on the Troposphere Collins model [14] inside a well known GNSS simulator called GPS-SDR-SIM written in *C* language and able to generates GPS baseband signal data streams, which can be converted to RF using software-defined radio (SDR) platforms. So, the simulation modeling is integrated with real equipment and real data elaborated by GPS receivers. In particular, the proposed module, implementing the Troposphere Collins model, is able to improve the accuracy of the simulation experiments.

The rest of this paper is organized as follows: Section 2 provides some related works about considered topic. In Section 4, a description of the GNSS applications in VANET scenario is given. In Section 5, we describe the used simulator considered for developing additional software module. The numerical results are presented in Section 6. Finally, Section 7 concludes the paper.

## 2. Related Work

Many works exist in the literature about GNSS software receivers. Starting from the GPS Standard Positioning Service, new systems have been proposed able to provide global coverage including GLONASS, Galileo and other regional satellite navigational systems, as described in Section 3. Many of these works concern software and hardware simulators with the aim of perform navigation tests in a secure and repeatable way.

### 2.1. GNSS Simulators

In the literature, several works have been proposed focusing on architectural and implementation aspects of GNSS simulators. In [15] the authors design a simulator of digital Intermediate Frequency (IF) signal based on the mathematical model of digital IF GPS signals. Their experiments show that the structure of simulated signals is closer to real signals. In [16] the authors propose a GPS I Galileo receiver called TUTGNSS. It provides a platform for testing and developing new algorithms for GNSS allowing developers to have full control over its further development. The open source Global Navigation Satellite System based on software-defined receiver, called GNSS-SDR, is proposed in [17]. The authors provide details about the software architecture design and implementation, for a multiband, multisystem GNSS receiver. They have built a testbed for customizable GNSS signal processing, able of permitting signal sources and signal processing algorithms management, guaranteeing systems and output format interoperability. In [18], the authors provide details about a GNSS software receiver implementation able to operate with Galileo E1B and E1C signals. They provide descriptions of the used algorithms that manage signals for navigation acquisition and tracking. An open source software implementation of a GPS receiver as a simple modification of a commercial-off-the-shelf (COTS) receiver is shown in [19]. In the paper, the authors describe the hardware and software architecture, the features added to allow debugging of the code and carrier tracking loops.

### 2.2. GNSS in VANET

Many works about GNSS in vehicular networks exist in the literature. Many researchers deal with the use of navigation system in applications typical of automative environment with the aim of perform navigation tests in a secure and repeatable way. In [7] the authors deal with the timing issue in VANET networks and then, on a GNSS-based time synchronization. They have conducted different experiments in order to test the accuracy of the time synchronization in a consumer-grade GNSS receiver. In [20] a novel unified Cooperative Positioning (CP) solution which enhances positioning accuracy and availability in urban canyons is presented. The proposal, called Angle Approximation (AA) does not require infrastructure or other aiding sensors and distributes and addresses two core challenges (limited positioning accuracy and availability) in a unified solution using a Dedicated Short Range Communication between vehicles. In [21] a vehicle localization enhancement solution inside VANET environment is addressed. Fusion of the GNSS and odometer measurement is augmented by ranging distance. A Dedicated Short Range Communication (DSRC) is used between vehicles. Experimental results show the performance of the proposed algorithm when GPS data is not temporarily available. In [22] the authors propose a novel local integrity concept for GNSS receivers in VANET environment. Their aim is to consider also the environment around the receiver in its nominal conditions and exploit the VANET infrastructure capability. In [23] the authors propose a new method based on a cooperative positioning in VANET in order to improve the relative positioning between two vehicles fusing the data of a GPS system.

### 2.3. Main Paper Contributions

In this work, we focused our attention on the absolute positioning system such as GPS, considering the simulation environment integrated with real devices in a vehicle scenarios. In particular, our main contribution regards:

- The introduction of a troposphere model in the GPS-SDR-SIM, an open-source simulator, in order to add the delay of the transmitted signal taking into account the troposphere.
- Co-simulation proposal where simulation modeling is integrated with real equipment and real data elaborated by GPS receivers. The proposed approach can be applied by researchers or industry to simulate positioning errors on realistic path working in the lab. and reducing performance evaluation costs.
- The goodness of the modeling proposal is proved by a set of simulation tests carried out at three different latitudes. It has been evaluated in terms of longitude and altitude errors.

## 3. Introduction to GNSS

Global Navigation Satellite Systems, abbreviated as GNSS is a term that refers to a number of multi-satellite systems that are owned, originated or being developed by different nations in the world and are (or would be) used to provide navigation and positioning data up to a certain level of accuracy for domestic, commercial, military and research purposes. Every GNSS has a constellation design that enables it to achieve a global coverage. The Global Positioning System (GPS)–USA, Galileo–Europe, GLObal’naya NAvigatsionnaya Sputnikovaya Sistema (GLONASS)–Russia, Compass Navigation Satellite System (CNSS)–China are the examples of GNSS. At the moment, GPS is the only GNSS which is fully operational. All other systems are either being developed or are partially operational. Table 1 shows difference parameters of various GNSS.

A GNSS is divided into three segments namely Space Segment, Ground Segment and User Segment. The space segment consists of the orbit constellation and the satellites (Space Vehicles). The ground segment consists of several monitoring stations on the ground, which send the corrected data about clocks and orbits to the satellites. The user segment consists of the GNSS receiver which can be of many different types.

### 3.1. Why Use GNSS Simulator

The applications utilizing GNSS technology is in constant increase. Many of these applications have strong requirements so it is necessary to integrate GNSS with other technologies to meet these requirements. To ensure that GNSS is reliable and guarantees reliable performance it is important that the development process is based on adequate tests from concept to production. In order to test a GNSS product three main methods are possible:

- Field tests using real-time signals.
- Registration test.
- Reproduction and laboratory simulation.

Today, best practices indicate that most tests are performed under controlled and repeatable conditions in a safe laboratory [24]. This allows testing of all conditions, including testing up to the real and theoretical performance limits. This also allows for the development of receivers for GNSS systems that are currently unavailable or lacking a complete constellation. Field testing of the real-world signal has significant disadvantages.

A summary of the benefits of testing with GNSS simulators, compared to live testing with true GNSS constellations, is listed below:

- Complete control over the signals from the constellations.
- Complete control over environmental conditions.
- Repeatability.
- No unwanted interference.
- No unwanted signal effects.
- Ability to easily test scenarios with GNSS constellation errors.
- Ability to test in the laboratory without involving vehicles.
- Ability to test constellations not yet present.

A GNSS simulator provides an effective and efficient mean of testing GNSS receivers, which will process simulated signals just like those from real GNSS satellites. It emulates the environment of a GNSS receiver on a dynamic platform by modeling the movement of the vehicle, satellite, signal characteristics, atmospheric effects and others, making the receiver actually navigate according to the parameters of the test scenario. Additionally, a GNSS simulator offers a superior alternative for testing, compared to using actual GNSS signals in a live environment. Unlike road tests, simulator tests provide full control of simulated satellite signals and simulated environmental conditions.

### 3.2. Pseudorange

The pseudorange represents the distance which the GNSS receiver measures between the satellite and the receiver’s antenna by measuring the travelling time, ΔT, the signal takes to propagate from the satellite (emission time) to the receiver (reception time) antenna. This value multiplied by the light speed gives the apparent range P=c·ΔT between them. This measurement P=c·ΔT is what we know as pseudorange or pseudodistance and it is an “apparent range” between the satellite and the receiver that does not match with its geometric distance due to, among other factors, synchronism errors between receiver and satellite clocks. Taking explicitly into account possible synchronism errors between these clocks, the travelling time between transmission and reception is obtained as a difference of times measured in two different clocks or time scales: the satellite ($t^{S}$) and the receiver ($t^{R}$).

## 4. GNSS Application in VANET

GNSS is considered an important component in the automative environment where the driving assistance is gaining always more attention in order to meet the requests of security and safety. The number of applications that use the GNSS technology is always higher and several applications have increasingly stringent requirements. So, in this context, the design of GNSS receivers is an important aspect to be considered. Traditionally, GNSS testing has been subdivided into following three distinct methods: Live sky testing, Record and Replay methods, Simulators. Nowadays, the best practices for testing GNSS receivers concern tests performed in a controlled and repeatable manner in a safe laboratory [24]. In such a way, it is possible to allow tests on the development of receivers currently unavailable or able to operate with a non-complete constellation. The satellite messages contain among other information the so–called ephemeris, in order to compute the satellite position at any time, allowing, with the corresponding distance estimations, the receiver to compute its own position and time. The distance between the receiver and a given satellite, $d_{i}$, can be so calculated:

(1) $$d_{i}=c\cdot \left(t_{i}^{R}-t_{i}^{S}\right),$$

where *c* is the speed of light, $t_{i}^{R}$ is the receiving time in the receiver’s clock, and $t_{i}^{S}$ the time of transmission for a given satellite *i*. Receiver clocks are inexpensive and not perfectly in synchronization with the satellite clock, and thus this time deviation is another variable to be estimated. In this field, the pseudorange term identifies a range affected by a bias. It refers to the clock error between the receiver and satellite. The main error is due to the signal propagation: the atmosphere introduces modification in the time because the difference between light and wave speed that results in an error on the distance estimation. The main task of a simulator is to emulate the different conditions of a GNSS receiver by modeling the satellite and vehicles behavior, the characteristics of the signal, the atmospheric effects and all relevant aspects concerning receiver navigation in the considered simulated environment. Unlike road tests, simulator tests provide full control of simulated satellite signals and simulated environmental conditions. In this way, testers can easily generate and run many different test scenarios for different types of tests, with complete control over all simulation parameters such as date, time position, vehicle movement and environmental conditions. The performance of a receiver varies on the basis of errors and effects applied to the RF signal. The evaluation of the simulation results can be done in real time or by post-test analysis of the recorded data by comparing receiver and simulation data.

### GPS-SDR-SIM: An Open Source Simulator for GPS Receivers

*GPS-SDR-SIM* [25] is an open source simulator written in *C* language that allows to generate baseband GPS data streams, which can be converted into RF signals using Software Defined Radio (SDR) platforms such as for example ADALM-Pluto [26] and HackRF One [27]. The software permits to specify the GPS constellation and, then, to generate the GPS signal based on a file called GPS broadcast ephemeris file. In this work, the file has been downloaded from the site [28] and a daily file containing information about the constellation at a given moment (in RINEX (Receiver Independent Exchange) format [29]) has been obtained. These files are then used to generate the simulated pseudorange and doppler frequency for the GPS satellites. These simulated data are then used to generate digitized I/Q samples for the GPS signal [30]. The simulation starting can be specified if the corresponding set of ephemeris is available, otherwise it is set on the basis of ephemeris present in the RINEX file. In addition to the RINEX file, a National Marine Electronics Association (NMEA) file (a text file describing the coordinates of the receiver in the form of strings) for the simulated vehicle coordinates has to be provided. These coordinates must have a frequency of 10 Hz, which means that 10 NMEA strings correspond to one second of simulation. A particular type of NMEA string specifies the position of the receiver; some of the most relevant parameters are—latitude, longitude, GPS signal quality, and so on.

The data is sent through messages (sentences) which differ in their content. Each sentence is composed of a starting character “$”, an ending character “*”, the checksum formed by two characters and finally by <CR> <LF>. After the $ character, two letters identify the device. In our satellite navigation case we have “GP” code that identifies GPS. The next three letters identify the type of message. Each message, in turn, has a predefined content divided into various fields separated by commas. The most common messages are: Global Positioning System Fixed Data (GGA), Recommended Minimum Specific GNSS Data (RMC), GNSS DOP and Active Satellites (GSA), GNSS Satellites in View (GSV), Geographic Position-Latitude/Longitude (GLL), Course Over Ground and Ground Speed (VTG). In particular, the NMEA string type for our experiments is GGA, Global Positioning System Fix Data. In the following, an example of NMEA string extract from our path in middle latitude and in Table 2 an explanation of the different terms in the string are provided:

$GPGGA,090000.00,3921.28520559,N,01613.51936448,E,1,05,2.87,160.00,M, −21.3213,M,,*7F

## 5. Tropospheric Model Solution

In this paper a model considering the signal delay crossing the troposphere space has been implemented into the software simulator.

**Figure 2:**
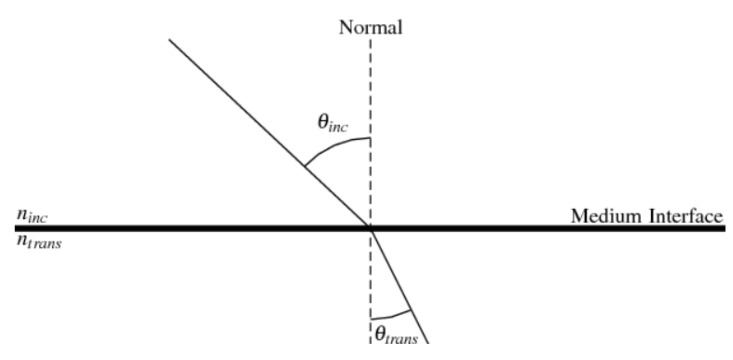
Example of the refraction phenomenon of the signal where $n_{inc}$ and $n_{tran}$ are the refractive indices of the incident and transmitted medium, respectively.

The troposphere is the lowest layer of the atmosphere and extends from the ground surface up to about 8 km at the pole and up to about 17 km at the equator. It contains about 75% of the total atmospheric mass. It is full of water vapor and varies in temperature and pressure. The troposphere is a refractive medium, which influences the GNSS signals propagation, see Figure 2. The troposphere induces an excess propagation path length on the GNSS signals given by the difference between the time taken by the signal to traverse the refractive medium, having a refractive index *n*, and the time it would have taken in vacuum conditions. Much of the delay caused by a signal’s trip through the atmosphere can be predicted. Atmosphere mathematical models consider different aspects regarding particles and gas composing the troposphere. In order to realize the purpose of the paper, a well-known model was selected to calculate the effects of the signal based on the parameters available in the simulator.

### 5.1. Tropospheric Collins Model

In this work the Troposphere Collins model [14] has been chosen for the implementation. This model is also used by Satellite-Based Augmentation System (SBAS) for maximum precision differential corrections, with the aim of increasing the accuracy and integrity of the GPS system data, such as the Wide Area Augmentation System (WAAS) and European Geostationary Navigation Overlay System (EGNOS), that are air navigation aids developed to augment the GPS, with the goal of improving its accuracy, integrity, and availability. The model provides an estimate of the zenith total tropospheric delay that is dependent on empirical estimates of five meteorological parameters at a receiver—namely, pressure, temperature, water vapour pressure, temperature lapse rate and water vapour lapse rate. These estimates of the meteorological parameters are dependent on the receiver’s height, latitude and day-of-year, and are interpolated from reference values for the yearly averages of the parameters and their associated seasonal variations.

The total tropospheric delay (indicated with T(E)) at a given elevation angle can be expressed as the sum of two different components of delay due to ‘wet’ and ‘dry’ delay:

(2) $$T\left(E\right)=\left(T_{z,dry}+T_{z,wet}\right)\cdot M\left(E\right),$$

where $T_{z,dry}$ is the zenith ‘dry’ delay, $T_{z,wet}$ is the zenith ‘wet’ delay and M(E) is a mapping function that maps the zenith total delay to the appropriate receiver to satellite elevation angle and it is defined as follows:

(3) $$M\left(E\right)=\frac{1.001}{\sqrt{0.002001+sin^{2}\left(E\right)}},$$

where *E* represents the satellite elevation with respect to the receiver, in degrees. The most difficult part of the model regards the estimation of the values of $T_{z,dry}$ and $T_{z,wet}$. These two values depend on meteorological parameters such as:

- Atmospheric pressure [*P* (mbar)].
- Temperature [*T* (K)].
- Water vapor pressure [*e* (mbar)].
- Lapse rate temperature [β (K/m)].
- Lapse rate of water vapor [λ (adimensional)]

Since these data are not available in the simulator, a formula was used to estimate them, based on various factors, such as: receiver latitude and day of the year:

(4) $$\xi \left(\phi ,D\right)=\xi_{0}\left(\phi \right)-\Delta \xi \left(\phi \right)cos\left[\frac{2\pi (D-D_{min})}{365,25},\right]$$

where $D_{min}$ assumes the value of 211 for the southern latitudes and the value of 28 for the northern latitudes. Values of $\xi_{0}\left(\phi \right)$ and Δξ(ϕ) represent the average seasonal variations at latitude (ϕ) and day of the year (*D*) of the receiver, which must be linearly interpolated with meteorological parameters given in Table 3 [14].

The terms $T_{z,dry}$ and $T_{z,wet}$ at zero altitude are the following:

(5) $$T_{z_{0},dry}=\frac{10^{-6}k_{1}R_{d}P}{g_{m}}$$

(6) $$T_{z_{0},wet}=\frac{10^{-6}k_{2}R_{d}P}{\left(\lambda +1\right)g_{m}-\beta R_{d}}\frac{e}{T}.$$

While, to calculate the delay taking into account also the height of the receiver, the following equations are used:

(7) $$T_{z,dry}={\left[1-\frac{\beta H}{T}\right]}^{\frac{g}{R_{d}\beta }}\cdot T_{z_{0},dry}$$

(8) $$T_{z,wet}={\left[1-\frac{\beta H}{T}\right]}^{\frac{(\lambda +1)g}{R_{d}\beta }-1}\cdot T_{z_{0},wet},$$

where *H* is the height of the receiver above sea level (m), $k_{1}$ = 77.604 (K/mbar), $k_{2}$ = 382,000 K${}^{2}$/mbar, $R_{d}$ = 287.054 J/Kg/K, $g_{m}$ = 9.784 m/s${}^{2}$, eg = 9.80665 m/s${}^{2}$.

### 5.2. Model Implementation

**Figure 3:**
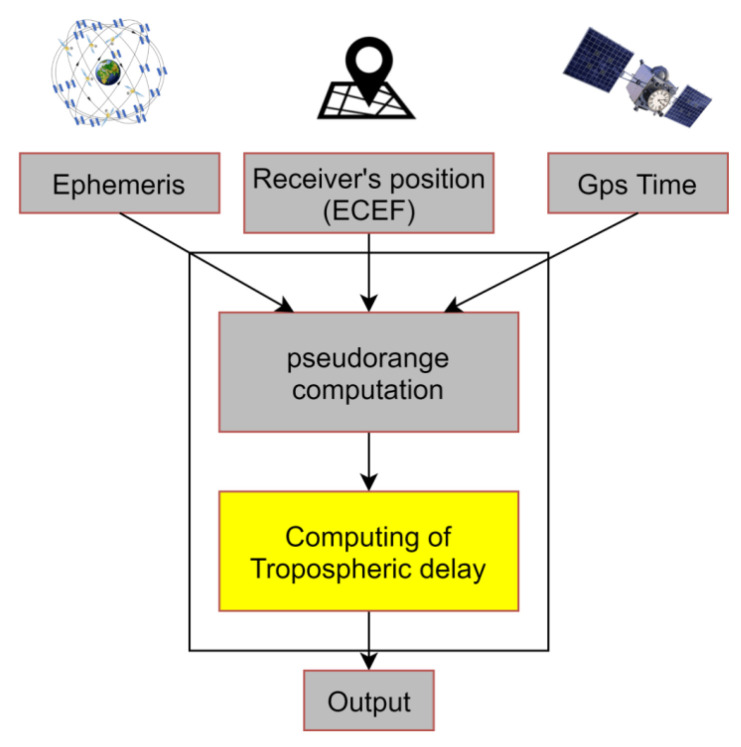
Tropospheric Block Diagram.

In Figure 3, a block scheme that summarizes implemented functionalities is shown.

In order to develop the model in the considered simulator, three functions have been created in *C* programming language able to calculate the signal delay, and then to add it in the pseudorange calculated for each satellite. The main method has been called *troposphericDelay*, see Algorithm 1, it takes as input the *g* variable of type *gpstime_t*, which is a struct that represents the GPS time in the week-seconds format; an array of three elements called *llh* which contains the latitude, longitude and altitude of the GPS receiver, and finally an array of two elements called *azel*, which indicates azimuth and elevation of the satellite with respect to the receiver, see the pseudocode of Algorithm 1.
**Algorithm 1:** troposphericDelay: Calculate the delay of the signal in meters due to the troposphere ( T(E) ).
 **Input**   : gpstime_t (output of gpsTimeToDays function, llh 
     (latitude, longitude, altitude), azel (azimuth and elevation of satellite)
 **Output**: T(E)
 1 Tz0dry;
 2 Tz0wet;
 3 calculate_Tz0dry;
 4 calculate_Tz0wet;
 4 T(E) = (Tzdry + Tzwet) * Me;
 5 **return** T(E);


The other two functions are:

- *gpsTimeToDays*: it has the aim of simply converting the GPS time from the week-seconds format, to the day of the year ranging from 1 to 365, the pseudocode of this method can be seen in Algorithm 2.
- *parametroTropos*: it has the aim of taking the listed values and, on the basis of the involved parameter such as latitude, satellite elevation and day of the year, it calculates the correct value, which will then be used in the main method, see Algorithm 3.

**Algorithm 2: **gpsTimeToDays: Converts from gpstime_t to the number of days from January.
 **Input**   : gpstime_t g
 **Output**: days from Jenuary
 1 weeknumber;
 2 seconds;
 3 days = weeknumber * 7;
 4 convert_g_in_daysFromJenuary(weeknumber,seconds,days);
 5 **return** daysFromJenuary;

**Algorithm 3: **parameterTropos: Calculate the parameter ξ as function of ϕ and *D*.
 **Input**  : Average Environmental Value (AEV): (P, T, e, b, l) which represent the coefficients relating to the environmental parameters tabulated in the Collins tropospheric model, based on the “latitude” (ϕ) of the receiver and the “day of the year” (D)
 **Output**: ξ(ϕ,D)
 1 $\xi_{0}\left(\phi ,D\right)$;
 2 Δξ(ϕ); $D_{min}$;
 3 calculate_ξ(AEV);
 4 **return**
ξ(ϕ,D);


## 6. Performance Evaluation

The conducted experiments are described in this section where, after showing the simulation environment, a discussion about results are given providing the simulator behavior extended by the Tropospheric Collins model.

### 6.1. Simulation Environment

**Figure 4:**
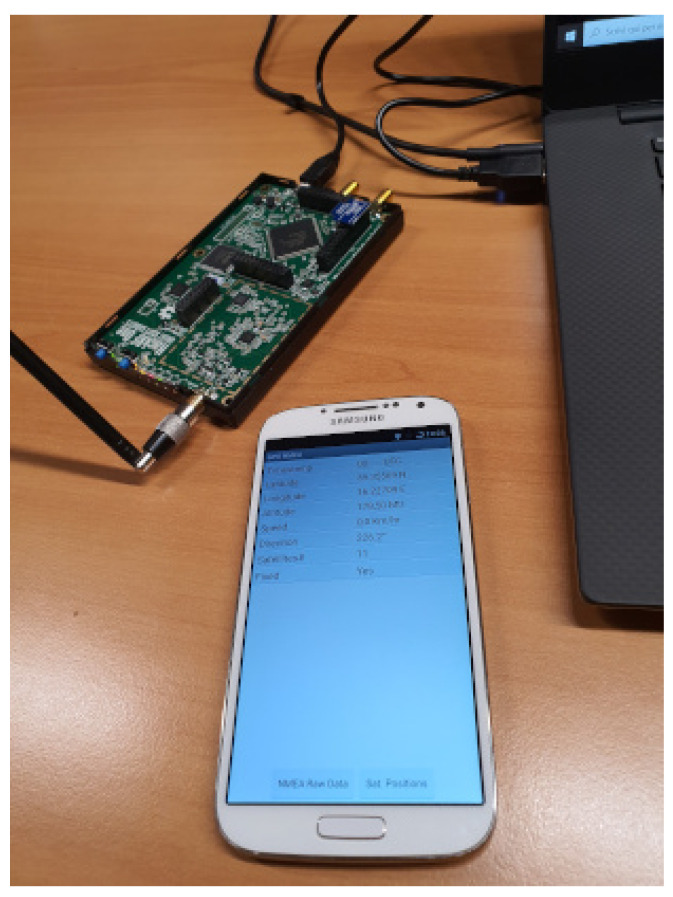
HackRF One with its ANT500 antenna and Tiny TCXO 10 Mhz oscillator and smartphone used as GPS receiver.

**Figure 5:**
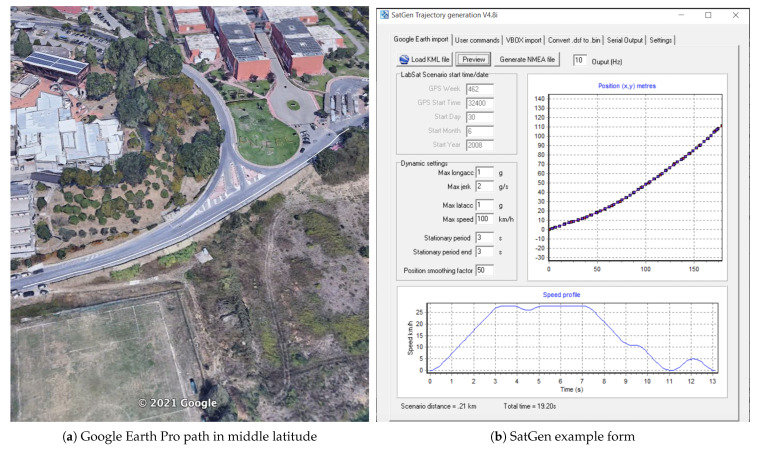
KML example file loaded with SatGen software.

A brief description of used hardware and software components are provided in the following. The sending device uses the HackRF One card with the ANT500 antenna and the Nooelec Module Tiny TCXO 10 Mhz module, a very precise oscillator with very low phase noise. It has been chosen from the various options available for the HackRF One card in order to use GPS applications (see Figure 4). An Android smartphone (Samsung Galaxy S4) with the NMEA GPS software was used as the receiving device, which returns the GPS data in NMEA format (see Figure 4). A path consisting of eleven points on the map and then converted into NMEA format, to be simulated with the *GPS-SDR-SIM* software has been created using Google Earth Pro software. From Google Earth Pro software, once a path has been choose, it is possible to generate a “KML” file which is then passed to another software: SatGen [31] in order to create the NMEA strings, see Figure 5. KML stands for Keyhole Markup Language is a file format used to display geographic data in an Earth browser such as Google Earth. The software interface allows to set in the form “Dynamic settings” some parameters regards the dynamic of the receiver by setting information about different accelerations and max speed that we have set to 100 km/h. Moreover, in the first graphic it is possible to view the path created in Google Earth Pro and in graphic below the speed profile of the receiver. The update rate for the route can be defined from 1 Hz to 100 Hz, this will defined the granularity of the route created in the software. We have set it to 10 Hz. This means to create 10 NMEA strings to each second.

**Figure 6:**
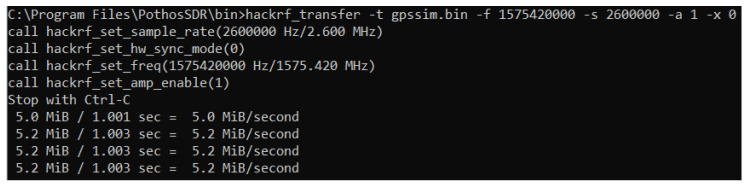
Command used to transfer the binary file to the HackRF One card.

Subsequently, the simulator output file has been transferred in input to the HackRF One board using Windows 10 command as shown in Figure 6.

The GPS signal is transmitted by the HackRF One board towards the smartphone GPS receiver deactivating/activating the troposphere correction algorithm. The experimental results are given in the next section and they show tests made considering three different latitude coordinates.

### 6.2. Experimental Results

The results of the conducted experiments are shown now in order to discuss the goodness of the proposal. The line called “real” path, colored in red, is related to the real coordinates given in input to the simulator.

**Figure 7:**
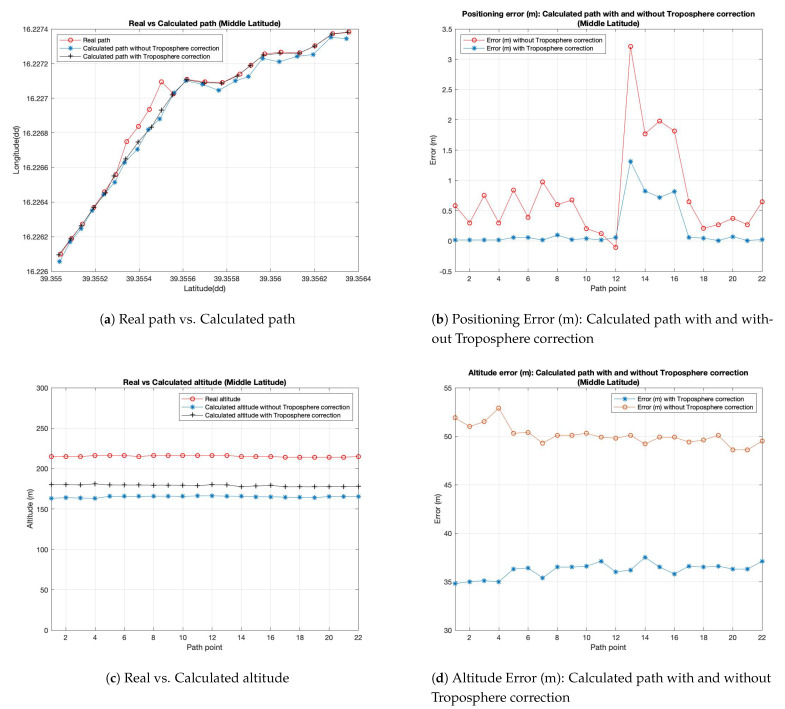
Experimental results for Middle Latitude (route in Italy).

In Figure 7a it is possible to observe a comparison between the “real” path and the two simulated ones calculated on the GPS receiver (the smartphone in the vehicle in our case): coordinates given in input to the simulator are depicted in red. This red line represents the reference for the other two lines representing the coordinates (in decimal degree (dd)) calculated by GPS receiver: the blue line (with the marker ‘*’) indicates the use of simulator with correction algorithm disabled, and the black one (with the marker ‘+’) indicates the use of the correction algorithm activated. GPS receiver should not deviate too much from points of red line in order to guarantee a correct GPS operation and, then a correct position. The figure shows that, in this case, the difference between curves representing the calculated path with and without troposphere correction is very small and not appreciable in this graphic, but the details about real error in meters is appreciable in the Figure 7b. Moreover, in order to show better the results and differences between the considered approach, we have increased path points extending the considered path in the three scenarios. The graphic in Figure 7b shows the positioning error of the two simulated paths in meters in respect to the real considered path. We precise that the positioning error represents the error committed by the calculated path with and without troposphere correction module considering the distance in meters between the coordinates of calculated path in respect to the real path. These two graphics show the results obtained starting from a path located at a middle latitude and an altitude of about 200 m above sea level. The graphic in Figure 7c shows the error committed in calculating the altitude above the sea level by the receiver. As it is possible to observe in this figure, the error on latitude and longitude is on average lower in the case in which the tropospheric algorithm is activated, for each point of the considered path. The goodness of the proposal has been assessed also for different latitudes: an equatorial latitude by a route in Brazil and an extreme latitude with a route in Norway have been considered and extrapolated by Google Earth Pro software.

**Figure 8:**
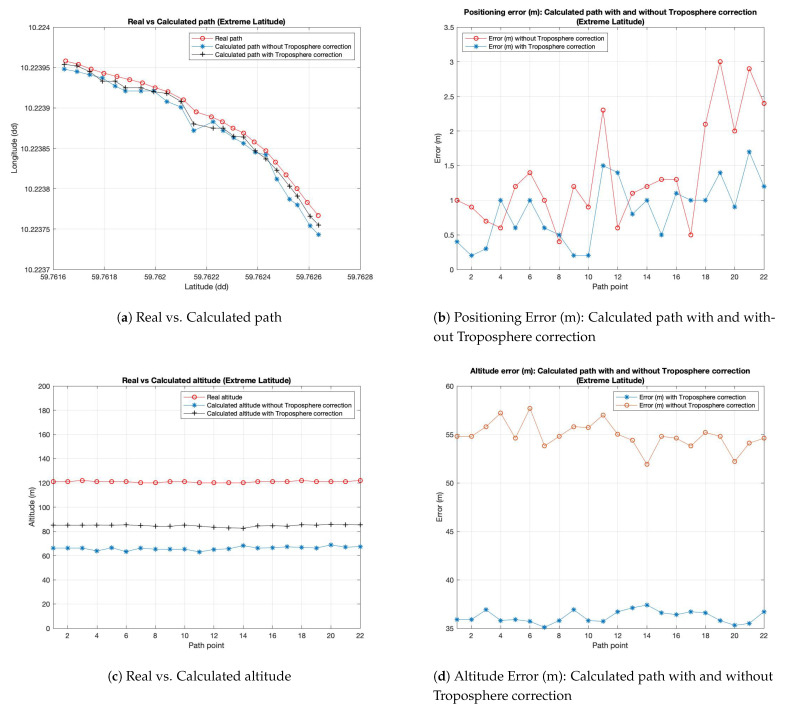
Experimental results for Extreme Latitude (route in Norway).

Figure 8a–c show the same output parameters illustrated above (in the case of middle latitudes) considering the extreme latitude coordinates. The same consideration, made for the previous case, is possible to do also for this new simulative campaign. It is possible to observe a better behavior of the system when the proposed module is activated in respect to the one with troposphere module disabled.

**Figure 9:**
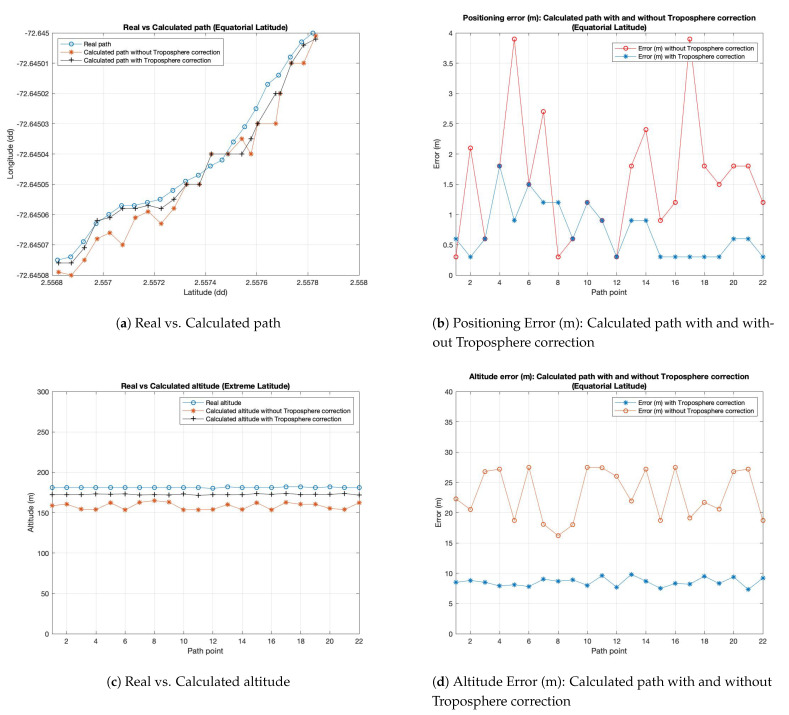
Experimental results for Equatorial Latitude (route in Brazil).

The third campaign is related to experiments considering equatorial latitudes. As it is possible to observe in Figure 9a–c, the results show how the obtained curves have the same trend of the previous cases. Comparing the graphics of the different campaigns, it is possible to observe a better behavior of the system at equatorial latitudes in respect to extreme ones. The graphics show an error lower in the equatorial case. Instead, taking into account extreme latitudes, an higher difference between the “real path” curve in respect to the simulated one (using the troposphere model) is possible to observe.

**Figure 10:**
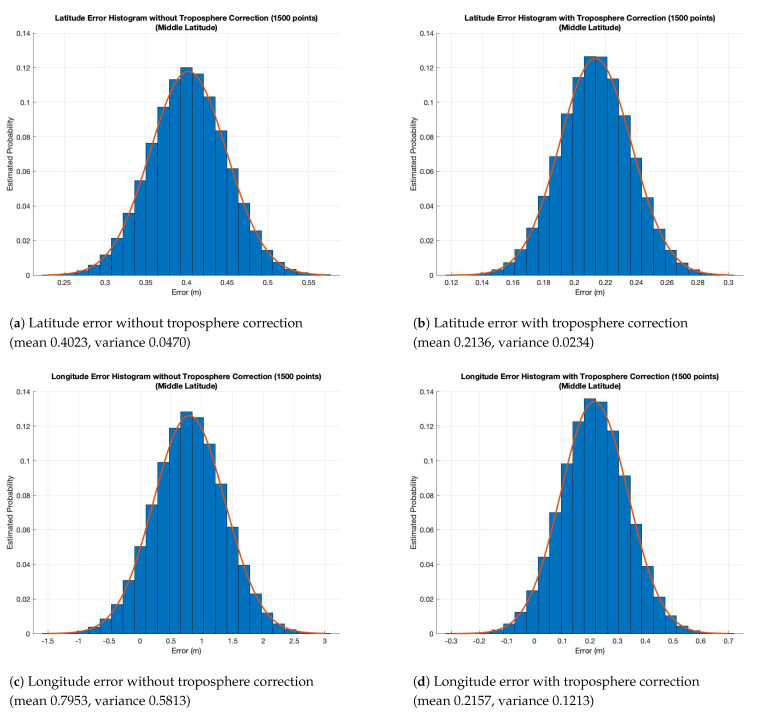
Histograms of experimental results on Middle Latitude.

Finally, in Figure 10 we present the estimated probability density functions, the histograms for a considered Middle Latitude experiments on a path of 1.8 km and taking into account 1500 path points. The graphics show that the errors distribution are very similar to the normal/Gaussian distribution. These figures prove that the model is correctly representing a GPS signal and, in particular, they show how the error committed by simulator considering the proposed module implementing the Collins model is lower than considering experiments without the troposphere correction module. In particular, Figure 10a represents the estimated probability density function for the latitude error committed without troposphere correction. It is possible to note a error range between about 0.25 to 0.55 m, with a mean of 0.4023 m and a variance of 0.0470 m. Figure 10b shows the latitude error range for experiments with the troposphere error compensation. In this case, it is possible to observe a lower error range with value between 0.12 and 0.3 m, with a mean of 0.2136 m and a variance of 0.0234 m. Instead, Figure 10c,d represent the estimated probability density function for the longitude error committed without troposphere correction and with troposphere correction, respectively. It is possible to note an error range between about −1.5 to 3 m, with mean of 0.7953 m and variance of 0.5813 m in the first case and an error range with value between −0.3 and 0.7 m, with mean of 0.2136 m and variance of 0.1213 m for the second one.

## 7. Conclusions and Future Works

In this paper, the introduction of a troposphere Collins model inside an open source GNSS simulator—called *GPS-SDR-SIM*—for a vehicles environment has been considered. The model introduces an additional signal delay (in form of pseudorange) in order to consider the effects of the troposphere for a more realistic scenario. The experiments have been conducted considering a specific hardware—HackRF One with its original ANT500 antenna, a very precise 10MHz nooelec oscillator and a smartphone as a GPS receiver placed in vehicles. Three different tests have been carried out considering three different latitude coordinates. The results show how the implemented tropospheric software module introduces an improvement in the system guaranteeing better performance in term of position errors. The introduction of the troposphere model allows to have errors in latitude/longitude coordinates and in altitude values lesser than those performing experiments with the *GPS-SDR-SIM* simulator without the correction module.

For the future works, it could be important to analyze the accuracy positioning characterizing the channel with an appropriate model, such as, for example, using approach based on markovian process [32,33].

## Figures and Tables

**Table 1:** Comparison of various GNSS.

	GPS	GLONASS	Galileo	CNSS
Country	USA	Russia	Europe	China
Number of Satellite	32	24	30	35
Number of orbital planes	6	3	3	10${}^{2}$
Orbital height (km)	20.200	19.140	23.222	21.150
Orbital period	11 h 58 m	11 h 15 m	14 h 06 m	12 h 48 m
Orbital Inclination	55${}^{{}^{\circ}}$	64.8${}^{{}^{\circ}}$	56${}^{{}^{\circ}}$	55.5${}^{{}^{\circ}}$
Coding	CDMA	FDMA	CDMA	CDMA
Carrier Frequencies (MHz)	1575	1559–1592	1579	1590
	1228	1243–2063	1279	1561
	1176		1207	1269
			1192	1207
			1176	1192

**Table 2:** NMEA GGA string description.

Value	Description
GPGGA	GP: GPS-GGA: type of message
Time format	hhmmss.ss (h: hours, m: minutes, s: seconds
090000.00	09 (h): 00 (m): 00.00 (s)
3921.28520559,N	$39^{{}^{\circ}}$$21.28520559^{\prime }$ North (latitude)
01613.51936448,E	$16^{{}^{\circ}}$$13.51936448^{\prime }$ East (longitude)
1	GPS Quality indicator
	0 = Invalid
	1 = GPS fix
	2 = DGPS fix
	3 = Fix GPS PPS
	4 = RTK (Real Time Kinematic) entire
	5 = RTK float
	6 = Navigazione Stimata (dead reckoning)
	7 = Input Manual
	8 = Simulation
05	number of satellites
2.87	Horizontal Dilution of Precision (HDOP)
	(This is a unitless number indicating how accurate the horizontal position is. Lower is better).
160.0,M	Altitude (meters above mean-sea-level)
−21.3213,M	Geoidal Separation, meteres: the difference between the $WGS84^{*}$ earth ellipsoid surface and mean-sea-level (geoid) surface, “-” = mean-sea-level surface below WGS-84 ellipsoid surface (* World Geodetic System 1984)

**Table 3:** Average environmental values.

		**Average**			
**Latitude(**°**)**	$P_{0}$ **(mbar)**	$T_{0}$ **(** ${}^{{}^{\circ}}$ **K)**	$e_{0}$ **(mbar)**	$\beta_{0}$ **(** ${}^{{}^{\circ}}$ **K/m)**	$\lambda_{0}$
15	1013.25	299.65	26.31	6.30 $\times \;10^{-3}$	2.77
30	1017.25	294.15	21.79	6.05 $\times \;10^{-3}$	3.15
45	1015.75	283.15	11.66	5.58 $\times \;10^{-3}$	2.57
60	1011.75	272.15	6.78	5.39 $\times \;10^{-3}$	1.81
75	1013.00	263.65	4.11.	4.53 $\times \;10^{-3}$	1.55
		**Seasonal variation**		
**Latitude (${}^{{}^{\circ}}$)**	**ΔP (mbar)**	**ΔT (${}^{{}^{\circ}}$K)**	**Δe (mbar)**	**$\Delta \beta_{0}$ (${}^{{}^{\circ}}$K/m)**	**$\Delta \lambda_{0}$**
15	0.00	0.00	0.00	0.00 $\times \;10^{-3}$	0.00
30	−3.75	7.00	8.85	0.25 $\times \;10^{-3}$	0.33
45	−2.25	11.00	7.24	0.32 $\times \;10^{-3}$	0.46
60	−1.75	15.00	5.36	0.81 $\times \;10^{-3}$	0.74
75	−0.50	14.50	3.39	0.62 $\times \;10^{-3}$	0.30

## Data Availability

Data sharing is not applicable to this article.

## Abbreviations

The following abbreviations are used in this manuscript:

AEV	Average Environmental Value
COTS	Commercial-Off-The-Shelf
EGNOS	European Geostationary Navigation Overlay Service
GGA	Global Positioning System Fixed Data
GLL	Geographic Position—Latitude/Longitude
GLONASS	Global’naya Navigatsionnaya Sputnikovaya Sistema
GNSS	Global Navigation Satellite System
GPS	Global Positioning System
GSA	GNSS DOP and Active Satellites
GSV	GNSS satellites in view
KML	Keyhole Markup Language
ITS	Intelligent Transportation System
NMEA	National Marine Electronics Association
RINEX	Receiver Independent Exchange
RMC	Recommended Minimum Specific GNSS Data
SBAS	Satellite-Based Augmentation System
SDR	Software Defined Radio
VANET	Vehicular Ad-Hoc Network
VTG	Course Over Ground and Ground Speed
WAAS	Wide Area Augmentation System
